# A jsPsych touchscreen extension for behavioral research on touch-enabled interfaces

**DOI:** 10.3758/s13428-024-02454-9

**Published:** 2024-07-12

**Authors:** Younes Strittmatter, Markus W. H. Spitzer, Nadja Ging-Jehli, Sebastian Musslick

**Affiliations:** 1 Department of Cognitive, Linguistic, and Psychological Sciences, Brown University, Providence, USA; 2 Department of Psychology, Martin-Luther University Halle-Wittenberg, Halle, Germany; 3 Institute of Cognitive Science, Osnabrück University, Osnabrück, Germany

**Keywords:** Online experiments, Dot-motion kinematogram, Touchscreen experiments

## Abstract

Online experiments are increasingly gaining traction in the behavioral sciences. Despite this, behavioral researchers have largely continued to use keyboards as the primary input devices for such online studies, overlooking the ubiquity of touchscreens in everyday use. This paper presents an open-source touchscreen extension for jsPsych, a JavaScript framework designed for conducting online experiments. We additionally evaluated the touchscreen extension assessing whether typical behavioral findings from two distinct perceptual decision-making tasks – the random-dot kinematogram and the Stroop task – can similarly be observed when administered via touchscreen devices compared to keyboard devices. Our findings indicate similar performance metrics for each paradigm between the touchscreen and keyboard versions of the experiments. Specifically, we observe similar psychometric curves in the random-dot kinematogram across the touchscreen and keyboard versions. Similarly, in the Stroop task, we detect significant task, congruency, and sequential congruency effects in both experiment versions. We conclude that our open-source touchscreen extension serves as a promising tool for data collection in online behavioral experiments on forced-choice tasks.

**Supplementary Information:**

The online version contains supplementary material available at 10.3758/s13428-024-02454-9.

## Introduction

Crowd-sourced online experiments have become increasingly popular across the behavioral sciences, including cognitive psychology, social psychology, and behavioral economics. The success of online experiments can be partly attributed to the ability to collect larger data sets within shorter periods compared to lab-based experiments. Despite vast differences in conditions under which experiments are performed online versus in the lab, several studies demonstrated that the results of online experiments are comparable to those of lab-based experiments (Crump et al., 2013; Buhrmester et al., 2011; Ramsey et al., 2016; Hilbig, 2016; de Leeuw & Motz, 2016; de Leeuw, 2015; de Leeuw et al., 2023; Semmelmann & Weigelt, 2018; Germine et al., 2012; Barnhoorn et al., 2014; March, 2021; Ratcliff & Hendrickson, 2021). In this study, we introduce a tool designed to expand the input options to include touchscreen interfaces, a technology with which people are becoming increasingly familiar (Ahearne et al., 2016; Neumann & Neumann, 2014).

On a practical level, online experiments have been facilitated by the development of open-source software for building online experiments, such as jsPsych (de Leeuw, 2015; de Leeuw et al., 2023). An important aspect of jsPsych is its support for novel plugins and extensions developed by the scientific community to enrich its functionality (e.g., Rajananda et al., 2018; Barnhoorn et al., 2014; Kinley et al., 2022; Donhauser and Klein, 2022; Gibeau, 2021; Callaway et al., 2017; Kuroki, 2021; Galang et al., 2021; Strittmatter et al., 2023). Despite its wide application, the jsPsych framework for conducting online experiments is mostly limited to collecting behavioral data from keyboard or mouse input, without general support for touchscreen experiments.

Evaluating data garnered from touchscreen devices pre-sents distinct advantages over traditional data collection methods using desktop computers or laptops without touch capabilities. Specifically, these advantages include: (a) broadening the pool of potential experiment participants, particularly given the more widespread use of touchscreen devices over keyboard-based platforms (StatCounter, 2016), (b) facilitating more ecologically valid research settings, owing to the enhanced portability and flexibility of mobile devices, (c) pioneering novel experimental paradigms optimized for touchscreens, and (d) acquiring richer, possibly longitudinal, data sets with greater ease, as touchscreen devices are readily accessible throughout the day.

Offline touchscreen experiments have already been administered across different species, such as rodents (Cook et al., 2004; Morton et al., 2006; Bussey et al., 1994, 2008; authorname, year; Dumont et al., 2021), monkeys (Hopper et al., 2021; Huskisson et al., 2020; Roy et al., 2000; Amiez et al., 2003), and humans (Robinson & Brewer, 2016; Atkinson, 2008; Clark et al., 2006) for decades. Moreover, behavioral data acquisition via touchscreens has gained popularity for studying human behavior (Bignardi et al., 2021; Pahor et al., 2022; Lacroix et al., 2021). For instance, Bignardi et al. (2021) administered app-based touchscreen experiments to 7 to 9-year-old children to examine cognitive abilities, such as visual search speed or arithmetic fluency. Their results indicate a high split-half reliability for touchscreen experiments. Further substantiating the robustness of such experiments, a study by Pronk et al. (2020) examined the timing accuracy of touchscreen responses through external validation mechanisms, such as brightness sensors for stimulus onset and solenoids for capturing participant responses^1^. Their results suggest that the timing accuracy of touchscreens is comparable to that of keyboard devices, provided that rapid stimulus presentations are circumvented (a minimum presentation time of 100 ms is recommended). However, Pronk et al. (2020) compared two smartphones (iPhone 6S and Samsung Galaxy S7) against two laptops (MacBook Pro and ASUS laptop). Nicosia et al. (2023) investigated the variability in response times exclusively across 26 popular smartphones. They found considerable variance between touchscreen devices in display and touch latencies, providing evidence that differences in general response times exist between touchscreen devices. Nevertheless, in the context of computational modeling, Gomez et al. (2015) have demonstrated that behavioral model parameters obtained from assessments of human behavior via touchscreens experiments are aligned with model parameters obtained from keyboard experiments (Gomez et al., 2015), demonstrating the comparability of results between touchscreen and traditional keyboard devices. Finally, a study by Lacroix et al. (2021) focused on evaluating visuospatial abilities through touchscreen interfaces. The research established strong correlations between data gathered via touchscreens and traditional paper-pencil methods, thereby validating the efficacy of touchscreen-based measures for assessing visuospatial skills.

In addition to existing validation studies on offline touchscreen interfaces, recent work began to compare online touchscreen measures with online keyboard measures (Passell et al., 2021; Pronk et al., 2023). For instance, Passell et al. (2021) compared participants’ general response times during web-based experiments on touchscreen and keyboard devices. They observed generally slower response times for touchscreen devices compared to keyboard devices. In another between-subject study with age-matched groups, Pronk et al. (2023) investigated whether the flanker effect (i.e., the RT difference between congruent and incongruent trials) is modulated when administered via touchscreen (smartphones) devices compared to keyboard devices. Their results indicate a flanker effect independent of the device group, with a significantly larger flanker effect for the smartphone group compared to the keyboard group. However, the variance in the effect did not differ across both groups. In line with the findings from Passell et al. (2021), response times were generally slower for the smartphone group compared to the keyboard group. As such, recent findings suggest that data obtained from touch-enabled devices like smartphones and tablets can replicate cognitive interference effects. However, response times should be generally slower when data is collected from touchscreen devices compared to keyboard devices.

**Fig. 1:**
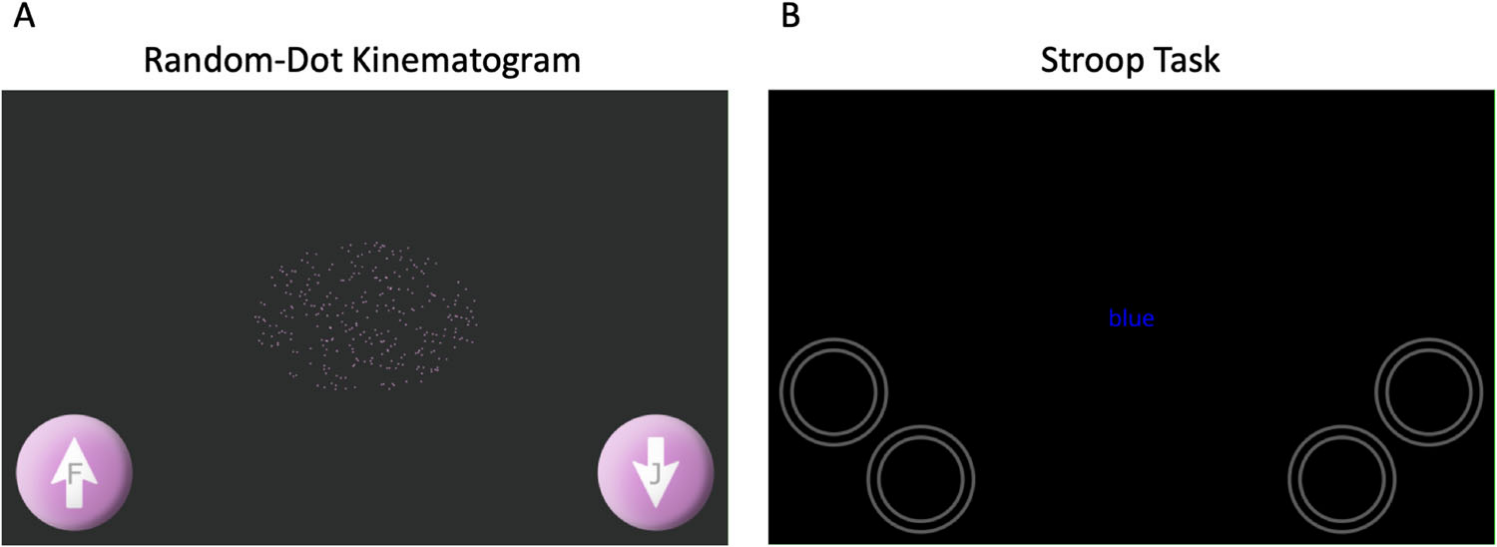
Interfaces for Studies 1 and 2. *Note*. Random-dot kinematogram stimulus (**A**) and a congruent Stroop stimulus (**B**). The stimulus presentation did not differ between the touchscreen and the keyboard version, except for the RDK touchscreen version, which had no “F” and “J” letters within the arrows. To avoid target-response congruency effects, we did not label or colorize the buttons in the Stroop task

In this study, our primary aim is to introduce a touchscreen extension for the jsPsych framework that seamlessly facilitates the conversion of keyboard-based experiments into their touchscreen equivalents. In addition, we also evaluate the efficacy of this extension through two experimental paradigms: the random-dot kinematogram and the Stroop task. By contrasting the data acquired from touchscreen implementations with their keyboard counterparts, we aim to assess the construct validity of canonical behavioral effects observed from two distinct perceptual decision-making tasks. The purpose of this evaluation is to demonstrate that data obtained from online touchscreen experiment versions of two distinct perceptual decision-making tasks reveal similar findings compared to data obtained from online keyboard experiment versions. Note that the sample size of this attempt was estimated based on previous findings on typical effects observed from the two paradigms but was not estimated to obtain potential differences in the effect size between devices. In other words, we seek to find similar qualitative effects across both device modalities, without considering a sample size that allows us to make robust inferences about differences in effect size. Our findings reveal similar behavioral outcomes from both versions, implying that the touchscreen extension stands as a promising tool for data collection in online experiments.

## jsPsych touchscreen plugin

Our touchscreen interface is written as an extension for jsPsych, a JavaScript framework for online experiments (e.g., see Fig. 1). The touchscreen extension, along with its documentation, is available at: https://github.com/jspsych/jspsych-con-trib/tree/main/packages/extension-touchscreen-buttons. It is published under the MIT License, it is open source, and is free of charge. It can be used in conjunction with other jsPsych plugins, replacing traditional key presses on keyboards with presses on touch buttons^2^.

The extension simulates keypresses when the user touches previously defined buttons on the screen. When the user touches a touchscreen button, a JavaScript keypress event is triggered. This functionality facilitates compatibility with other jsPsych plugins that rely on keypresses for user responses. However, it is not advisable to integrate this extension with plugins that utilize other methods to record user responses, such as plugins that natively rely on touch inputs or plugins with text input since the extension is not compatible with these plugins and can not emulate other responses than keypresses. Furthermore, the current implementation does not support the recording or triggering of events based on motion-related touch interactions, such as pinching or swiping. Consequently, the extension exclusively captures click responses and does not accommodate the collection of data pertaining to motion-specific touch gestures, which may be desired for certain types of experiments.

To use the extension, the user initializes jsPsych with it. In the initialization step, various layouts can be configured and named. For example, a layout for instructions screens with a single continue button and a layout for the stimulus presentation with two or more response buttons. Each layout can feature an arbitrary amount of buttons. Since the touchscreen buttons emulate keypresses, keys have to be assigned to each touchscreen button. This is the only setting that is obligatory. Additional settings include the styling of the buttons. The user can either choose from a set of predefined settings or customize the position and styling of the buttons. For easy use, we provide simple default settings for the position, size, and color of the button. However, the user can also fully customize the button by using all CSS properties that are native to online applications. The following example shows a use case with two different layouts (for a comprehensive description of all the settings, see https://github.com/jspsych/jspsych-contrib/tree/main/packages/extension-touchscreen-buttons).

Here, we initialize jsPsych with two different layouts. The instructions layout features a single button at the bottom of the screen (for example, to use as a continue button on instructions), and the stimulus-presentation layout features two buttons on the left and right side of the screen that with the text ”left“ and ”right“. 
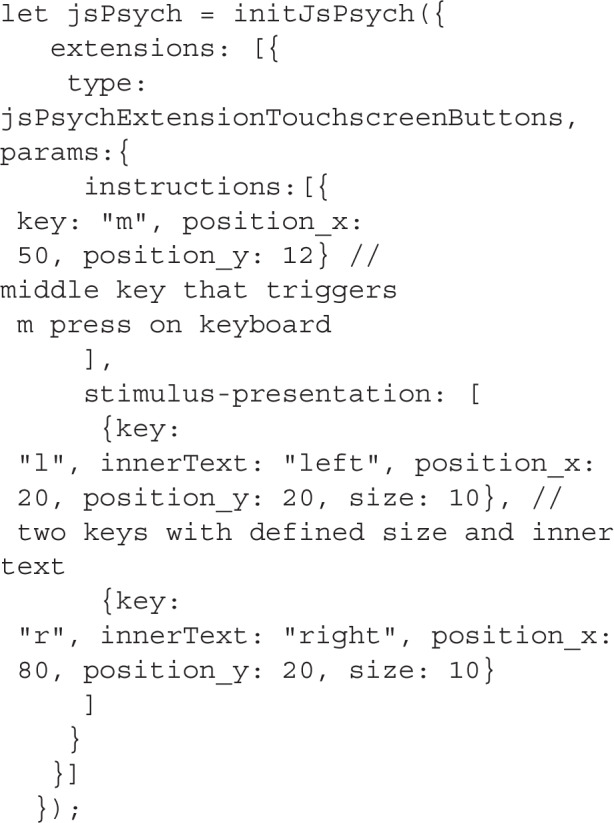


Experiments designed with jsPsych comprise of trials. Here, we show a text that prompts the user to press a button. To integrate the extension into a jsPsych trial, add the extension to the trial’s specifications and define the layout. The process remains consistent with that of trials without the extension. The extension is compatible with all plugins that accept keyboard input. Note that the choices parameter in the trial needs to match the keys assigned in the initialization step of the extension: 
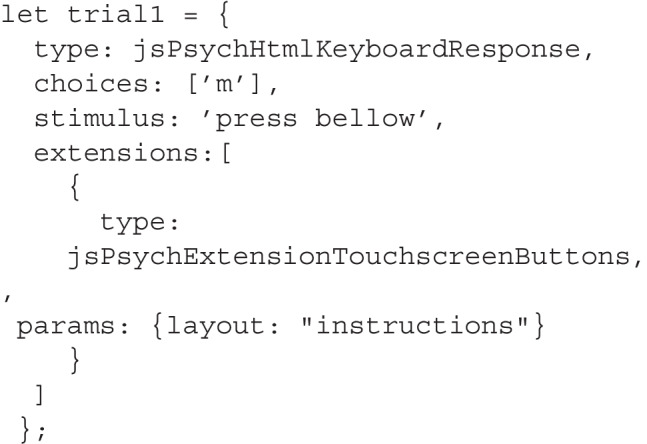


If we want to use a different layout, we have to pass in the layout name as a parameter for the touchscreen extension: 
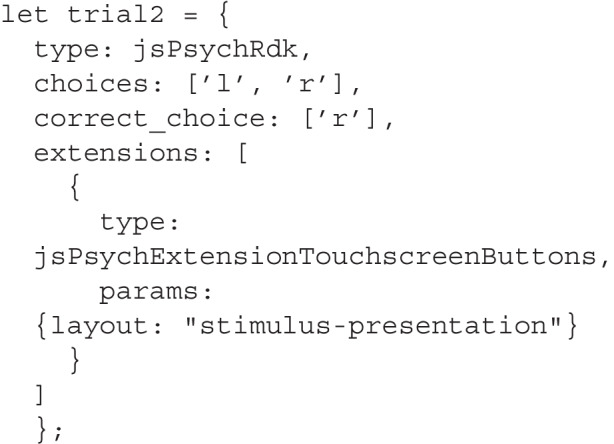


Users can provide feedback on the plugin, suggest changes, request features, or report bugs on the jsPsych contributor repository https://github.com/jspsych/jspsych-contrib/tree/main/packages/extension-touchscreen-buttons.

To examine the reliability of our extension, we compare data from online touchscreen versions and online keyboard versions of the same experiment across two paradigms. The next sections outline two experimental studies, which examine the tool within a random-dot kinematogram (Study 1), as well as the Stroop task (Study 2).

## Study 1: Random-dot kinematogram

The random-dot kinematogram has become a popular tool for the study of decision-making in two-alternative forced choice tasks (Kayser et al., 2010; Mante et al., 2013; Shadlen & Newsome, 1996; Newsome & Pare, 1988; Newsome et al., 1989; Shadlen & Newsome, 2001). The random-dot kinematogram consists of dots that move in a random direction, with some proportion of the dots moving coherently up or down. In paradigms involving this stimulus, participants are typically asked to indicate the direction most of the dots are moving.

A pivotal feature of the random-dot kinematogram is its flexibility in adjusting coherence levels, which can range from 100% coherence – where all dots move uniformly in one direction – to 0%, where each dot moves randomly. This enables the construction of a psychometric curve, illustrating the relationship between coherence levels and behavioral metrics like reaction time (RT) and accuracy. Such curves have been well documented in previous studies (Baker et al., 1991; Lankheet & Verstraten, 1995), reporting a non-linear increase in accuracy, saturating at chance performance (i.e., 50% in two-alternative forced choice tasks). Similarly, RT is found to decrease with increases in coherence (also see Strittmatter et al. (2023); Spitzer et al. (2022, 2024) for online administrations of the task).

To evaluate the touchscreen plugin, we examined psychometric curves in a random-dot kinematogram paradigm across two versions of the same online experiment, one using a touchscreen interface and one using a traditional keyboard. We manipulated coherence levels in 10% increments, ranging from 90% to 10%. To refine the resolution at the extremes, we also included additional coherence levels at 95% and 5%. This resulted in a spectrum of eleven coherence levels, allowing for a comprehensive mapping of the psychometric curves for both experiment version modalities regarding RT and accuracy. Building on previous work (Strittmatter et al., 2023; Spitzer et al., 2022, 2019), we expected a non-linear and saturating decrease in RT with increased coherence levels. Similarly, we expect an increase in accuracy with increasing coherence.

### Methods

#### Participants

We collected data from 36 participants (18 female participants; 18 male participants; *M*$_{age} = 27.33$; *SD*$_{age}= 3.21$) for the touchscreen version and from 38 participants for the keyboard version (19 female participants; 19 male participants; *M*$_{age} = 24.83$; *SD*$_{age}= 2.49$) using the Prolific recruitment platform. We chose the sample sizes based on previous studies examining psychometric curves in this paradigm (Strittmatter et al., 2023). In particular, we simulated a power analysis based on data from Experiment 1 of Strittmatter et al. (2023) using the simr package in R Green and MacLeod (2016). The power analysis was based on 34 participants, a beta of -.3, an alpha level of 0.05, and 100 simulations. This simulation revealed a power of 99% with a 95% confidence interval (95% CI) between 94 and 100%. Thus, we decided to oversample to account for any drop-outs and exclusions (see Results for details) and thus, collected data from a sample size of 38 participants per version. The data of two participants of the touchscreen version were not tracked due to technical reasons during data collection. Thus, this sample only included 36 participants. We did not conduct an a priori power analysis for potential differences between devices as the purpose of this evaluation was to show that typical effects that can be observed with keyboard devices can also be observed with touchscreen devices.

Participants earned $2.5 for participating in this study, which lasted approximately 20 min. Participants gave infor-med consent before the start of the experiment, prior to the task instructions. The participants for the touchscreen version performed the experiment on mobile devices with smartphones or tablets. Conversely, the participants for the keyboard version performed the experiment with desktop computers or laptops. We excluded participants from the touchscreen version from participating in the keyboard version and vice versa. The study received approval from the Institutional Review Board at Brown University.

#### Stimuli

We conducted the online random-dot kinematogram experiment with the jsPsych software (de Leeuw, 2015; de Leeuw et al., 2023) and applied the random-dot kinematogram with the rdk-plugin (Rajananda et al., 2018). Each stimulus consisted of 300 purple dots presented on a gray background (see Fig. 1 for a stimulus example) with a dot radius of 2px and a moving distance of 1px per frame. We used the same stimuli across both experiment versions.

#### Response interface

Both experiment versions displayed two response buttons, one at the lower left and the other at the lower right corner of the screen. The response buttons were colored purple to match the stimulus color. The buttons showed a white upward arrow and a white downward arrow indicating the responses for upward and leftward motions, respectively (see Fig. 1). Both experiment versions displayed the response buttons for the entire stimulus duration or until the participant responded.

The touchscreen version of the experiment instructed participants to indicate the coherent dot motion by touching the respective response button on the screen. The keyboard version of this experiment displayed the same buttons but instructed participants to indicate their responses via a key press. Participants were required to press the “F”-key to indicate upward-moving dots and the “J”-key to respond to downward-moving dots, and vice versa, depending on the counterbalancing scheme described below. In the keyboard version of the experiment, the response buttons displayed respective response keys in addition to the arrows.

#### Procedure

*Experiment blocks* Both experiment versions consisted of nine blocks. The first block was a training block with 20 trials. All subsequent eight blocks contained 44 trials each. We applied eleven coherence levels (95%, 90%, 80%, 70%, 60%, 50%, 40%, 30%, 20%, 10%, and 5%) in a random order within each block.

*Trial structure* In both experiment versions, each trial started with a fixation cross presented for 600 ms, followed by the RDK stimulus until the participant responded (or until a time limit of 2000 ms). After a correct response, participants received feedback presenting the word “CORRECT” for 500 ms. If the participant did not respond, “TOO SLOW” in red ink appeared for 500 ms. After incorrect responses, “INCORRECT” in red ink appeared on the screen for 500 ms.

*Counterbalancing* In both experiment versions, we counterbalanced the position of the response buttons across participants, e.g., in the touchscreen version, we showed the upwards arrow on the left side of the screen and the downwards arrow on the right side of the screen, and vice versa. In the keyboard version, we additionally counterbalanced the response-key mapping across participants, i.e., we instructed half of the participants to press “F” for upward motion and “J” for downward motion.

#### Variables

The movement coherence of the dots was treated as a continuous factor representing the independent variable of the experiment. We carried out separate analyses for RTs and accuracies, which served as dependent variables.

#### Data analysis

We performed all data analyses using the lmerTest package (Kuznetsova et al., 2017), the lme4 package (Bates et al., 2014), and the brms package (Burkner, 2015) in R (R Core Team, 2023) and plotted figures and tables using the sjPlot package (Lüdecke, 2020).

*Anticipated effects* We investigated the main effect of coherence on RT and accuracy for the touchscreen and keyboard experiment versions separately. For RT, we used a hierarchical linear regression model with which we regressed trial-wise RT against coherence. We log-transformed RT for the analysis as we assumed a non-linear relationship between coherence and non-transformed RT. For accuracy, we used a hierarchical logistic regression model to regress the trial-wise accuracy against the same independent variable. For both models, we modeled the coherence variable as a random slope to account for variability between participants regarding the effect of coherence. We implemented participants as a random intercept to account for between-participant variability in overall RT and accuracy. We observed, however, that the results were virtually identical when we did not model a random slope effect. We expected a main effect for the coherence with higher coherence, resulting in lower RT and lower accuracy.

*Response interface effect* After we showed that both versions exhibited the anticipated effects, we pooled the data from the touchscreen and keyboard versions to quantify whether significant differences between the response interfaces existed. Therefore, we ran the same hierarchical regression models with an additional response interface variable as the main and interaction effects. General RT or accuracy disparities (e.g., whether responses are consistently slower or faster, or more or less accurate in one version compared to the other) would be indicated by a main effect of the response interface. Differences related to how coherence affects responses between the two interfaces would be captured by an interaction between coherence and response interface. Note, however, that the sample size of the two experiment versions was estimated based on the effect of coherence on participants RT and was not estimated to investigate whether the device type modulated the effect of coherence (see General discussion for more in-depth elaboration on this point).

*Response interface and screen size effects* Touchscreen and keyboard devices may not only differ with respect to the response modality but also with respect to screen size, as screen sizes vary between smartphones, tablets, laptops, and desktops. Thus, the effect of coherence may not only be modulated by the response interface but also by the screen size. Therefore, we carried out another hierarchical regression model with the three factors coherence, response interface, and screen size as main effects including all interactions between the three terms.

*Null effect testing* We were particularly interested in showing a null effect for the coherence and response interface interaction. Thus, we ran additional Bayesian hierarchical regression analyses with the brms package in R Burkner (2015) to quantify whether the 95% confidence interval (CI) of the posterior distribution of our models’ estimates was around zero – providing evidence for a null effect. We estimated the two Bayesian hierarchical regression models with 500 warm-up samples, 3000 iterations, and four chains.

### Results

All participants responded with an accuracy above 60%, so we did not exclude any participant based on overall performance. We excluded trials with unreasonable fast RTs below 200 ms (touchscreen 1.7%; keyboard 0.9%). For the RT analyses, we excluded incorrect responses (touchscreen 13.8%; keyboard 14.9%). On average, participants in the touchscreen version of this experiment responded 44ms slower (average touchscreen RT 774 ms; average keyboard RT 739 ms) but had a similar accuracy (average touchscreen accuracy 86%; average keyboard accuracy 86%). For the touchscreen experiment version, most participants responded with smartphones (32 participants) and only four participants responded with tablets. Most participants used the Chrome browser in both experiment versions (see Fig. S4).

**Fig. 2:**
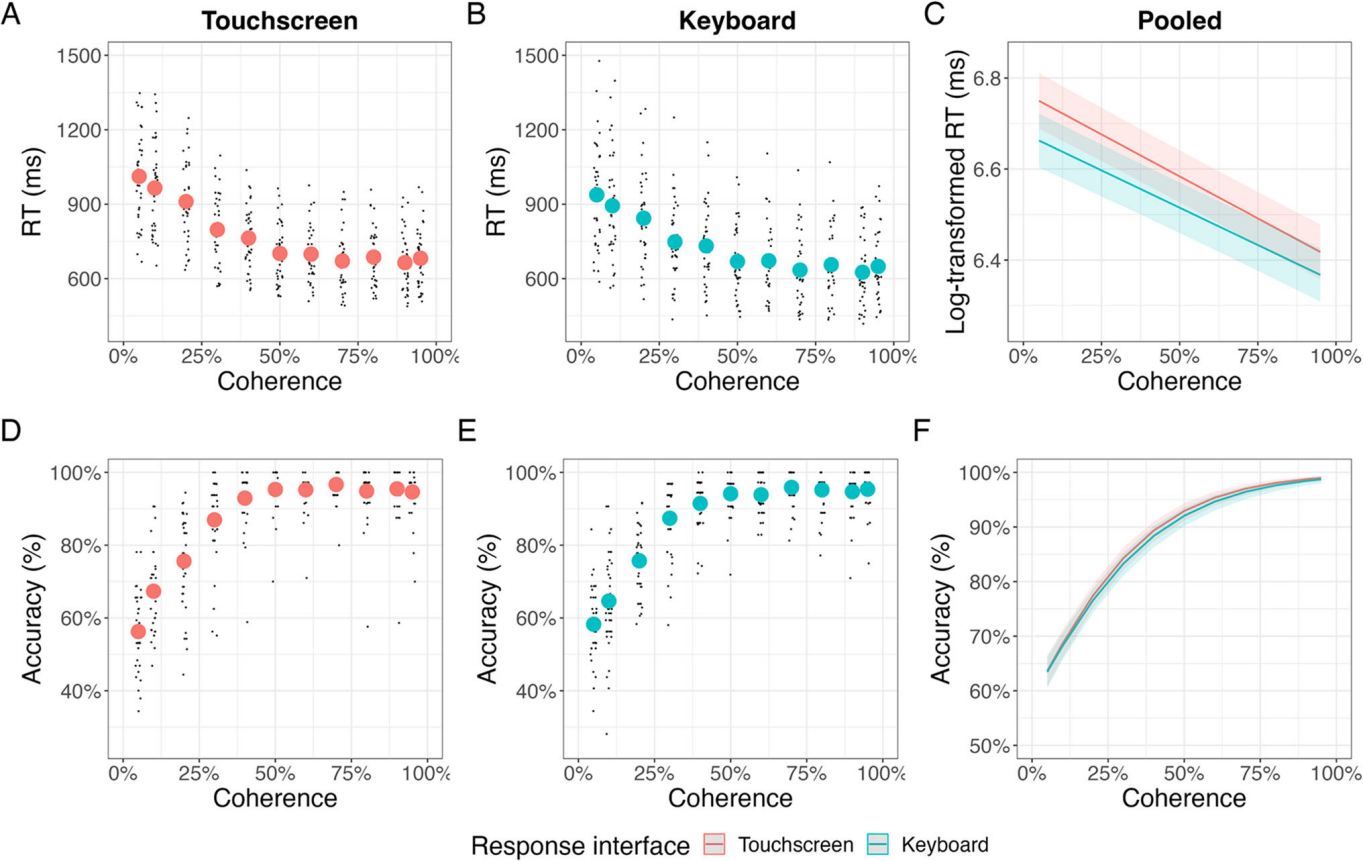
Study 1: Touchscreen, keyboard, and pooled hierarchical regression results for RT (**A**–**C**) and accuracy (**D**–**F**). *Note.* A–B The RTs decreased following an exponential psychometric curve with increasing coherence for the touchscreen version (**A**) and the keyboard version (**B**). **C** Log-transformed RT decreased with increasing coherence for the touchscreen and the keyboard version. RTs were faster for the keyboard version than for the touchscreen version. **D**, **E** Accuracies increased following an exponential psychometric curve with increasing coherence for the touchscreen version (**D**) and the keyboard version (**E**). **F** Accuracies increased with increasing coherence for the touchscreen and the keyboard version. *Red dots* (A, D) and *turquoise dots* (B, E) indicate averages over participants for each coherence. *Black dots* indicate the averages for each participant for each coherence. *Solid lines* (C, F)indicate the hierarchical regression fits. *Shaded areas* indicate the standard error of the mean

Figure 2 depicts the RT as a function of coherence and accuracy. Figure S1 depicts RT distributions for correct and incorrect responses for each coherence level for each version, respectively. The analysis with response interface and screen size revealed no significant effect of screen size on participants’ RTs and accuracy. In addition, the results indicated no modulatory effect of screen size with coherence. We report the results of this analysis in the Supplementary Material.

#### Reaction times

*Anticipated effects* In the touchscreen version, the RT significantly decreased with increasing coherence, as indicated with a significant coherence term (*b* = -0.37; *t* = -15.33; *p*< .001). We observed a similar pattern in the corresponding keyboard version (*b* = -.33; *t* = -15.24; *p* < .001). Figure 2 shows comparable psychometric curves for RTs across the touchscreen version (Fig. 2A) and the keyboard version (Fig. 2B) of the experiment.

*Response interface effect* Results of the hierarchical linear regression model are depicted in Fig. 2C and indicated a significant main effect for the coherence term (*b* = -0.35; *t* = -21.64; *p* < .001). The main effect for the response interface was significant (*b* = -0.04; *t* = -2.03; *p* = .046) and indicated that the RT was generally slower in the touchscreen version than in the keyboard version. The interaction between coherence and response interface was not significant (*b* = 0.02; *t* = 1.27; *p* = .21).

*Null effect testing* In line with the results from the hierarchical linear regression model on the pooled data reported above, we observed that the 95% CI of the posterior distribution for coherence was below zero (estimate = -0.35; lower 95% CI = -0.38; upper 95% CI = -0.32; Rhat = 1.00), indicating that RT decreased with increasing coherence. We also observed that the effect for the response interface was below zero (estimate = -0.04; lower 95% CI = -0.09; upper 95% CI = 0.00; Rhat = 1.00), indicating a slower RT in the touchscreen compared to the keyboard version. Finally, and most importantly, the 95% CI for the interaction between coherence and response interface was around zero, supporting the null effect (estimate = 0.02; lower 95% CI = -0.01; upper 95% CI = 0.05; Rhat = 1.00).

#### Accuracy

*Anticipated effects* In the touchscreen version, accuracy increased significantly with increasing coherence (*b* = 4.49; *t* = 15.04; *p*< .001). We observed the same effect for the keyboard response interface (*b* = 4.21; *t* = 15.99; *p* < .001). Figure 2 depicts the psychometric function for accuracy that appears comparable across the touchscreen version (Fig. 2D) and keyboard version (Fig. 2E) of the experiment.

*Response interface effect* As for the RT analysis, we pooled the data for both experiment versions for two further analyses on accuracy: a hierarchical logistic regression model with the response interface as an additional factor and the same model but with a Bayesian estimation approach.

The hierarchical logistic regression model results are depicted in Fig. 2F. We observed a significant effect for the coherence term, indicating increasing accuracy with increasing coherence (*b* = 4.35; *t* = 22.01; *p* < .001). The main effect for response interface (*b* = 0.01; *t* = 0.06; *p* = .953) and the interaction between coherence and response interface was not significant (*b* = -0.13; *t* = -0.67; *p* = .502).

*Null effect testing* The Bayesian model suggested that the 95% CI for coherence was outside of zero, indicating a positive effect of increasing coherence on accuracy (estimate = 4.35; lower 95% CI = 3.95; upper 95% CI = 4.80; Rhat = 1.00). In contrast, the 95% CI for the response interface (estimate = 0.00; lower 95% CI = -0.09; upper 95% CI = 0.10; Rhat = 1.00), indicating no overall accuracy differences between the response interface versions (supporting a null effect). In addition, the interaction between response interface and coherence was around zero (estimate = -0.14; lower 95% CI = -0.53; upper 95% CI = 0.27; Rhat = 1.00), also indicating no accuracy differences on the effect of coherence between the response interface.

### Discussion

As a first evaluation of the touchscreen interface, we examined behavioral performance across touchscreen and keyboard interfaces in the random-dot kinematogram in a between-subjects design. The objective was to determine if similar effects and psychometric curves could be seen when collecting data through touchscreens compared to keyboards. We found similar psychometrics across the touchscreen and keyboard interfaces, showing non-linear decreases in RT and increases in accuracy as a function of coherence (Fig. 2). Overall, the touchscreen and keyboard experiment versions showed similar effects of coherence on RT and accuracy. The sole divergence we noted was slightly higher RTs in the touchscreen version compared to the keyboard version. Overall, these outcomes underscore similar coherence effects between the touchscreen and keyboard experiment versions.

## Study 2: Stroop task

The Stroop task is a cornerstone experiment in cognitive psychology designed to explore the mechanisms underlying our ability to override habitual responses, collectively referred to as cognitive control (Cohen et al., 1990; Stroop, 1935). In this task, participants are presented with color words, such as “red” or “blue”, displayed in either matching or conflicting ink colors (Stroop, 1935). They are then tasked to either respond to the color word or the ink color, while participants are typically faster and more to the word (task effect). Another effect commonly observed is the congruency effect. The congruency effect indicates that participants generally respond more quickly and accurately when the color word and ink color match (i.e., congruent), as opposed to when they differ (i.e., incongruent) (Stroop, 1935). This disparity is attributed to cognitive interference, which occurs when the automatic tendency to read the word interferes with the deliberate effort to identify the ink color (Cohen et al., 1990). More nuanced phenomena are the task-congruency (Cohen et al., 1990) interaction and the sequential congruency effect (Gratton et al., 1992). The interplay between task and congruency effects leads to a heightened congruency effect (disparity between performance metrics on congruent compared to incongruent trials) in the color-naming task compared to the word-naming task (Cohen et al., 1990). Additionally, the congruency sequence effect indicates that the congruency of a preceding trial can modulate performance on the subsequent trial (Gratton et al., 1992). Specifically, incongruent trials are often processed more rapidly if they follow another incongruent trial, suggesting that cognitive control dynamically adjusts in response to the level of conflict encountered (Botvinick et al., 2001). Together, all these effects provide valuable insights into our ability to override habitual responses, also described as cognitive control.

Here, we investigate whether an online touchscreen version of a Stroop experiment yielded similar results to a corresponding keyboard version (Fig. 1). As with Study 1, we assigned different groups of participants to the different experiment versions and examined whether similar effects could be observed for the two experiment versions.

### Methods

#### Participants

For the touchscreen version of the experiment, we collected data from 48 participants (24 female participants; 24 male participants; *M*$_{age} = 28.13$; *SD*$_{age}= 3.41$). We collected data for another 48 participants for the keyboard version (24 female participants; 24 male participants; *M*$_{age}= 25.61$; *SD*$_{age} = 4.51$). The sample size was based on an a priori power analysis with G*Power (Faul et al., 2007) according to which a sample size of 44 participants should be sufficient to detect a medium effect size of d = .5 between two dependent means for an alpha error of 0.05 and power of 90%). Based on this analysis, we decided to collect data from 48 participants per group in case of dropouts. We assumed that the sample size should be sufficient to detect canonical effects of the Stroop task, such as the congruency effect, the interaction between task and congruency, and the sequential congruency effect^3^. Importantly, and similar to Study 1, our sample size was not calculated to detect potential differences in the observed effects observed in the Stroop task between devices.

We recruited participants for both experiment versions from the Prolific recruitment platform. All participants gave informed consent before the experiment prior to reading the instructions. Each experiment rewarded participants with $3 for their participation, which lasted 15 min on average. As in Study 1, the participants performed the touchscreen version of the experiment on mobile devices with smartphones or tablets, while participants in the keyboard version used desktop computers or laptops. We restricted participation to only one version of the experiment. The study received approval from the Institutional Review Board at Brown University.

#### Stimuli

The total set of stimuli encompassed the four words “RED”, “BLUE”, “YELLOW”, and “GREEN”, each presented in one of four colors: red, blue, yellow, and green. That is, color words were presented either in the same ink as the color word (congruent stimulus) or in a different ink (incongruent stimulus; see Fig. 1). As in Study 1, we leveraged the jsPsych software package (de Leeuw, 2015; de Leeuw et al., 2023) for stimulus presentation.

#### Response interface

Both experiment versions employed four circular response buttons. We presented two touch buttons in the lower left part of the screen and two in the lower right part of the screen (see Fig. 1). The buttons were unlabeled to not interfere with the word of the Stroop stimuli and were colored grey to not interfere with the color of the Stroop stimuli.

The touchscreen version of the experiment instructed participants to indicate their responses by touching the respective button on the screen. The keyboard version displayed the same buttons but instructed the participants to indicate their responses by pressing the keys “D”, “C”, “N”, and “J”. Unlike the buttons of the random-dot kinematogram in Study 1, we did not overlay the buttons with the respective letters. This decision was made because the buttons lacked any informative element, such as the arrow present in Study 1. The response-button mapping in the touchscreen version and the response-key mapping in the keyboard version were counterbalanced, as described below.

#### Procedure

*Experiment blocks* Both experiment versions consisted of eight blocks divided into two halves. In one half of the blocks, we asked the participants to respond to the color word of the Stroop stimuli (word task). In the other half, we asked them to respond to the ink color (color task). We counterbalanced the order of the tasks across participants. Each half of the experiment consisted of a training block with 194 trials, followed by three experimental blocks, each with 194 trials.

*Trial structure* Each trial began with a fixation cross displayed for 600 ms, followed by the Stroop stimulus until the participant responded (or until a time limit of 3000 ms was reached). The stimulus was followed by a blank screen, filling an inter-trial interval of 400ms. The stimulus was followed by feedback in the training block. The feedback displayed the words “CORRECT”, “INCORRECT”, or “TOO SLOW”, depending on whether the participants responded correctly, incorrectly, or failed to respond, respectively. We accompanied both “TOO SLOW” and “INCORRECT” with a reminder text showing the button-response mapping in the touch experiment and the key-response mapping in the keyboard experiment. The reminder text was shown for 4500 ms. The feedback was displayed for 500 ms. Trials were separated by an inter-trial interval of 400 ms, showing a blank screen. No feedback was given in the experimental block.

*Counterbalancing* We counterbalanced the crossed word and color factors for each experimental block, resulting in 25% congruent trials and 75% incongruent trials. Furthermore, we independently crossed three transition factors denoting a trial-wise switch or repetition of the color word (word transition), the ink color (color transition), and the stimulus congruency (congruency transition). Between subjects, we counterbalanced the positions of the buttons in the touchscreen version and the response-key mapping in the keyboard version.

**Fig. 3:**
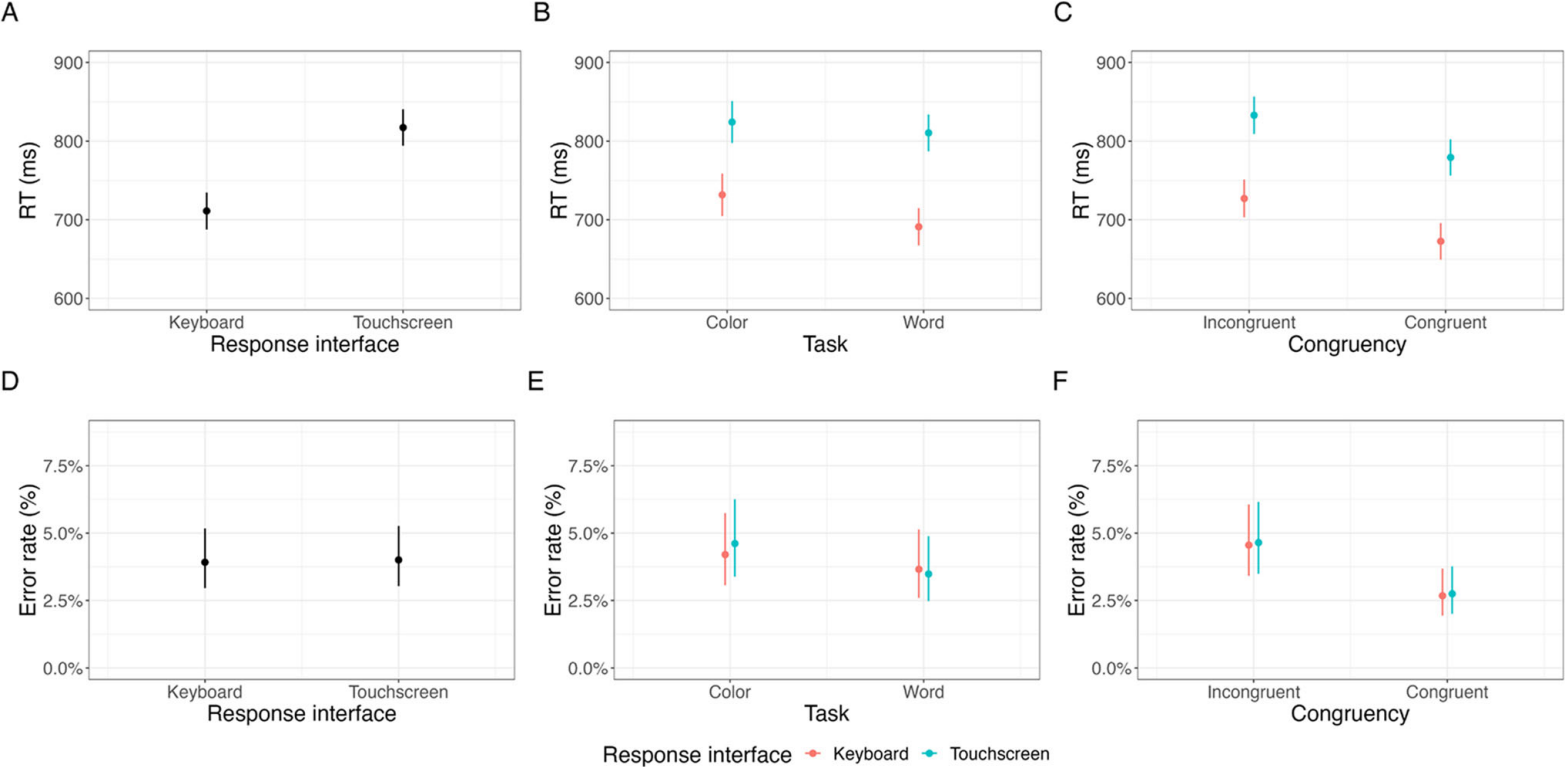
Study 2: Main effects of response interface, task, and congruency

#### Variables

Three categorical factors comprised our independent variables: (i) a task factor indicating whether participants respon-ded to the word or the color; (ii) a stimulus congruency factor indicating whether the color and word were congruent (e.g., the word “RED” in red ink) or incongruent (e.g., the word “RED” in blue ink); (iii) a previous congruency factor indicating whether the trial preceding the current trial was congruent or incongruent. In contrast to Study 1, we chose to present the error rate instead of accuracy in the present study. This decision aligns with the prevailing convention of reporting error rates in studies on the Stroop task (Cohen et al., 1990). Additionally, it facilitates a more direct comparison between the effects on RTs and error rates, given that changes in both RT and error rates indicate analogous performance differences. As in Study 1, we carried out separate analyses for RTs and error rates, which served as dependent variables.

#### Data analysis

As in Study 1, We used R (R Core Team, 2023) and performed the analyses using the packages lmerTest (Kuznetsova et al., 2017), lme4 (Bates et al., 2014), the brms (Burkner, 2015). We visualized the results using the sjPlot package (Lüdecke, 2020).

For both Stroop experiment versions, we did not collect data on screen size, browser type, device type, and operating system. We therefore were not able to analyze the effect of screen size on participants’ RTs and accuracy.

*Anticipated effects* We first regressed trial-wise RT and error rate against the three independent variables as main effects and their interactions, using a hierarchical linear regression model. We included the task and congruency factors as random slopes to account for between-participant variability for each factor. We did not include the interaction effects as random slope variables due to the resulting complexity of the model. We also opted not to include the previous congruency factors as a random slope, as including the factor as a random slope did not improve the model fit. However, the results reported below were virtually identical to a model that included the three factors as random slope effects. We included participants as a random intercept term in the hierarchical linear regression model to account for variability in RT between participants. Finally, we examined the error rate using a hierarchical logistic regression model with the occurrence of an error as the binominal dependent variable and the same three main effects, random slopes, and random intercepts.

We expected a main effect for the task factor reflected in slower responses and more errors in the color task than in the word task. We expected a congruency effect with faster responses and fewer errors on congruent compared to incongruent trials. We expected a significant interaction between the task and congruency factors, resulting in a greater congruency effect in the color task compared to the word task. Finally, we expected a sequential congruency effect, reflected in a decreased congruency effect on trials following incongruent compared to congruent trials.

*Response interface effect* After we showed that both versions exhibited the anticipated effects, we tested whether the two response interfaces significantly differed by pooling the data of both response interfaces and running the same hierarchical linear regression with the additional response interface factor as the main effect and interaction effect with all other variables.

*Null effect testing* As in Study 1, we were interested in showing a null effect for the response interface interactions. Therefore, we ran a Bayesian hierarchical regression analysis with 500 warm-up iterations, 3000 iterations, and four chains (as in Study 1) to quantify whether the 95% CI of the non-significant interactions centered around zero reflects evidence for a null effect.

### Results

**Fig. 4:**
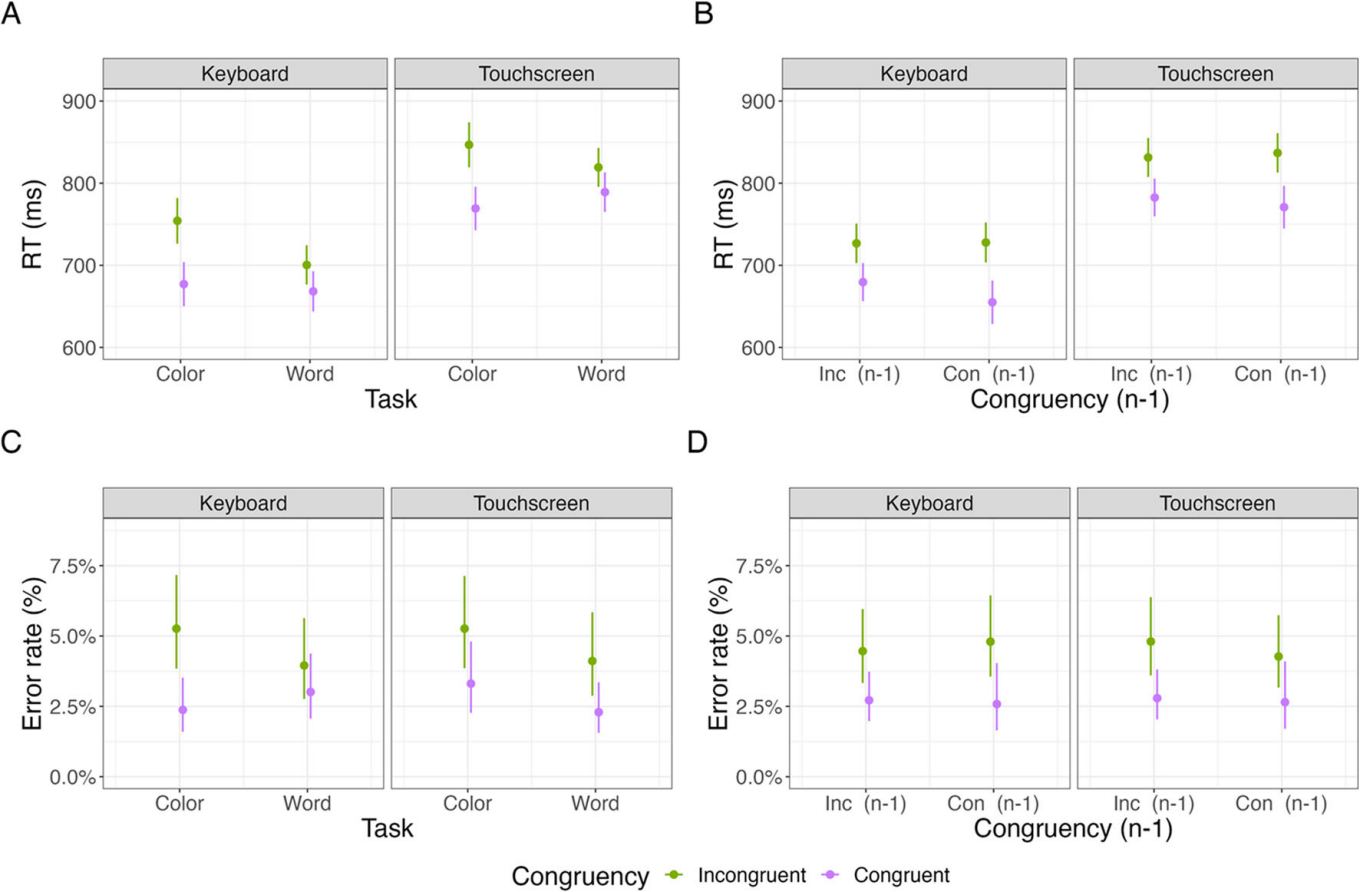
Study 2: Interaction effects between task, congruency, previous congruency, and response interface

Figures 3 and 4 show the results of Study 2. We did not exclude any participants from the touchscreen version of this experiment, as all participants had an error rate below 40%. In the keyboard version, we excluded two participants as their error rate was above 40%. In both experiment versions, we excluded trials with an RT below 200ms (touchscreen 4.8%; keyboard 1.0%). For the RT analyses, we excluded trials with incorrect responses (touchscreen 6.4%; keyboard 5.1%).

**Table 1:** Study 2: Hierarchical linear regression on RTs

	Touchscreen				Keyboard			
Coefficient	b	std. Error	*z*-value	*p* value	b	std. Error	*z*-value	*p* value
Intercept	805.23	11.62	69.32	**<0.001**	697.29	12.24	56.98	**<0.001**
Task	−1.38	5.34	−0.26	0.796	−15.35	5.02	−3.06	**0.002**
Congruency	−28.79	2.67	−10.78	**<0.001**	−30.18	2.18	−13.85	**<0.001**
Congruency (n-1)	−1.54	2.01	−0.77	0.443	−5.88	1.98	−2.97	**0.003**
Task: Congruency	11.41	2.01	5.67	**<0.001**	11.65	1.98	5.88	**<0.001**
Task: Congruency (n-1)	0.65	2.01	0.32	0.748	0.34	1.98	0.17	0.864
Congruency: Congruency (n-1)	−4.31	2.01	−2.15	**0.032**	−6.4	1.98	−3.23	**0.001**
Task: Congruency: Congruency (n-1)	−0.97	2.01	−0.48	0.628	0.88	1.98	0.44	0.658

*Note.* Estimates (betas), 95% standard errors, t-values, and *p* values of the hierarchical linear regression model for RTs in the touchscreen version and the keyboard version of the Stroop paradigm

#### Reaction times

*Anticipated effects* Table 1 shows the results of the hierarchical linear regression concerning RTs. We observed that the main effect for the task was not significant in the touchscreen but significant in the keyboard version of this experiment, with slower responses in the color task compared to the word task in the keyboard version(touchscreen *b* = -1.38; *t* = -0.26; *p* = .797; keyboard *b* = -15.34; *t* = -3.06; *p* = .003).

The main effect for congruency was significant in both experiment versions, with about 30ms faster responses on congruent compared to incongruent responses in both experiments (touchscreen *b* = -28.79; *t* = -10.77; *p* < .001; keyboard *b* = -30.17; *t* = -13.84; *p* < .001).

The main effect for previous congruency was not significant in the touchscreen but significant in the keyboard version, with faster responses on previous congruent compared to previous incongruent responses in the keyboard version (touchscreen *b* = -1.54; *t* = -0.77; *p* = .443; keyboard *b* = -5.88; *t* = -2.97; *p* < .003).

The interaction between task and congruency was significant in both experiment versions with an increased congruency effect in the color task compared to the word task (touchscreen *b* = 11.41; *t* = 5.67; *p* < .001; keyboard *b* = 11.65; *t* = 5.87; *p* < .001).

**Table 2:** Study 2: Bayesian hierarchical regression on RTs

	Estimate	Est. Error	l 95% CI	u 95% CI	Rhat	Bulk ESS	Tail ESS
Intercept	750.87	8.79	733.64	767.62	1.01	541.18	1265.79
Task	−8.50	3.76	−15.98	−1.22	1.00	1464.96	2563.53
Congruency	−29.48	1.76	−33.02	−26.08	1.00	5253.45	6847.61
Congruency (n-1)	−3.72	1.40	−6.47	−0.99	1.00	7522.00	7560.04
Response Interface	53.65	8.47	37.31	70.15	1.01	563.34	1242.03
Task: Congruency	11.54	1.42	8.76	14.31	1.00	7377.52	7826.90
Task: Congruency (n-1)	0.50	1.40	−2.25	3.26	1.00	6386.48	6989.06
Congruency: Congruency (n-1)	−5.38	1.40	−8.16	−2.67	1.00	7335.25	7657.71
Task: Response interface	6.77	3.77	−0.65	14.16	1.00	1358.14	2584.84
Congruency: Response interface	0.65	1.75	−2.68	4.21	1.00	5231.42	6204.42
Congruency (n-1): Response interface	2.15	1.41	−0.62	4.92	1.00	8129.46	7472.91
Task: Congruency: Congruency (n-1)	−0.06	1.40	−2.78	2.71	1.00	6338.01	6896.16
Task: Congruency: Response interface	−0.09	1.41	−2.85	2.66	1.00	8015.72	7727.95
Task: Congruency (n-1): Response interface	0.15	1.43	−2.68	2.89	1.00	8476.96	7821.51
Congruency: Congruency (n-1): Response interface	1.04	1.41	−1.71	3.79	1.00	7499.74	6995.38
Task: Congruency: Congruency (n-1): Response interface	−0.93	1.42	−3.71	1.85	1.00	7293.99	7617.61

*Note.* Estimates, Est. Error, lower 95% CI, upper 95% CI, Rhat, Bulk ESS, and Tail ESS of the Bayesian hierarchical regression model for RTs in the touchscreen version and the keyboard version of the Stroop paradigm

The interaction between previous congruency and congruency was significant in both experiment versions, with a reduced congruency effect after incongruent trials (in n-1) compared to congruent trials (in n-1) (touchscreen *b* = -4.31; *t* = -2.15; *p* = .03; keyboard *b* = -6.40; *t* = -3.23; *p* = .001).

The two-way interaction between task and previous congruency was not significant in both experiment versions(touchscreen *b* = 0.64; *t* = 0.32; *p* = .748; keyboard *b* = 0.34; *t* = .172; *p* =.863) and the three-way interaction was also not significant (touchscreen *b* = -0.97; *t* = -0.48; *p* = .628; keyboard *b* = 0.87; *t* = 0.44; *p* = .657).

*Response interface effect.* We observed a significant main effect for the task, with slower responses on the color task than the word task (*b* = 8.57; *t* = -2.35; *p* = .021); congruency, with faster responses on congruent than incongruent trials (*b* = -29.47; *t* = -17.05; *p* < .001); previous congruency, with faster responses following congruent trials than following incongruent trials (*b* = -3.72; *t* = -2.63; *p* = .008); and response interface, with slower responses when responding with a touchscreen than with a keyboard (*b* = 54.01; *t* = 6.44; *p* < .001). We also observed a significant interaction between congruency and previous congruency (*b* = -5.36; *t* = -3.79; *p* < .001). None of the other interactions were significant. Importantly, none of the interaction effects with the response interface were significant.

*Null hypothesis testing.* With respect to the response interface, the results indicated slower responses with the touchscreen interface than with the keyboard interface (see Table 2). The 95% CI for the interaction between the response interface and any of the other variables was around zero, suggesting that the response interface did not modulate the effect of task, congruency, or previous congruency (see Table 2).

**Table 3:** Study 2: Hierarchical logistic regression on error rates

	Touchscreen				Keyboard			
Coefficient	b	std. Error	*z*-value	*p* value	b	std. Error	*z*-value	*p* value
Intercept	−3.34	0.16	−21.02	**<0.001**	−3.3	0.15	−22.4	**<0.001**
Task	−0.18	0.12	−1.57	0.116	−0.04	0.08	−0.53	0.595
Congruency	−0.28	0.07	−3.96	**<0.001**	−0.29	0.07	−4.29	**<0.001**
Congruency (n-1)	−0.05	0.05	−0.92	0.358	0.01	0.05	0.14	0.891
Task: Congruency	−0.05	0.05	−0.94	0.346	0.13	0.05	2.56	**0.01**
Task: Congruency (n-1)	−0.03	0.05	−0.54	0.59	−0.07	0.05	−1.45	0.147
Congruency: Congruency (n-1)	0.02	0.05	0.35	0.728	−0.03	0.05	−0.63	0.529
Task: Congruency: Congruency (n-1)	−0.01	0.05	−0.3	0.761	−0.03	0.05	−0.65	0.516

*Note.* Estimates (betas), 95% standard errors, *z*-values, and *p* values of the hierarchical logistic regression model for error rates in the touchscreen and keyboard versions of the Stroop paradigm

**Table 4:** Study 2: Bayesian hierarchical regression on error rates

	Estimate	Est. error	l 95% CI	u 95% CI	Rhat	Bulk ESS	Tail ESS
Intercept	−3.33	0.11	−3.55	−3.11	1.00	1002.43	1827.15
Task	−0.11	0.07	−0.25	0.04	1.00	2555.80	3991.11
Congruency	−0.29	0.05	−0.39	−0.19	1.00	5240.32	6519.19
Congruency (n-1)	−0.03	0.04	−0.09	0.04	1.00	7476.85	8100.10
Response interface	0.01	0.11	−0.22	0.22	1.01	1154.99	2630.00
Task: Congruency	0.04	0.04	−0.03	0.12	1.00	7131.31	7957.92
Task: Congruency (n-1)	−0.05	0.04	−0.12	0.02	1.00	7154.71	7496.52
Congruency: Congruency (n-1)	−0.01	0.04	−0.08	0.05	1.00	7547.33	7709.50
Task: Response interface	−0.07	0.07	−0.21	0.08	1.00	2127.18	3619.96
Congruency: Response interface	0.01	0.05	−0.08	0.11	1.00	5180.49	6741.55
Congruency (n-1): Response interface	−0.02	0.04	−0.09	0.04	1.00	8009.33	7364.46
Task: Congruency: Congruency (n-1)	−0.02	0.04	−0.09	0.04	1.00	6857.18	8025.58
Task: Congruency: Response interface	−0.08	0.04	−0.15	−0.01	1.00	7594.50	8175.41
Task: Congruency (n-1): Response interface	0.02	0.04	−0.05	0.09	1.00	7723.45	7710.59
Congruency: Congruency (n-1): Response interface	0.03	0.04	−0.04	0.09	1.00	7908.65	7123.53
Task: Congruency: Congruency (n-1): Response interface	0.01	0.04	−0.06	0.08	1.00	7481.46	7725.06

*Note.* Estimates, Est. error, lower 95% CI, upper 95% CI, Rhat, Bulk ESS, and Tail ESS of the Bayesian logistic regression model for error rates in the touchscreen and keyboard versions of the Stroop paradigm

#### Error rates

*Anticipated effects.* Table 3 shows the results of the hierarchical logistic regression concerning error rates. We observed no significant effect of the task factor in both experiment versions, albeit participants exhibited a higher average error rate on the color task as compared to the word task (touchscreen *b* = -0.18; *t* = -1.57; *p* = .116; keyboard *b* = -0.04; *t* = -0.53; *p* = .595).

The main effect for congruency was significant in both experiment versions (touchscreen *b* = -0.27; *t* = -3.96; *p* < .001; keyboard *b* = -0.29; *t* = -4.29; *p* < .001), with lower error rates on congruent compared to incongruent responses.

The main effect for previous congruency was not significant in both experiment versions (touchscreen *b* = -0.04; *t* = -0.92; *p* = .358; keyboard *b* = 0.01; *t* = 0.13; *p* = .890).

The interaction between task and congruency was not significant in the touchscreen but significant in the keyboard version of this experiment, with an increased congruency effect for the color task compared to the word task (touchscreen *b* = -0.27; *t* = -3.96; *p* < .001; keyboard *b* = 0.13; *t* = 2.56; *p* = .01). None of the other interactions were significant.

*Response interface effect.* As for the RT analysis, we computed a hierarchical logistic regression model with a response interface as a factor. We observed two significant effects. First, a significant main effect for coherence (*b* = -0.28; *t* = -5.76; *p* < .001), indicating lower error rates on congruent trials. Second, we observed a significant interaction between task, congruency, and response interface (*b* = -0.08; *t* = -2.18; *p* =.028), indicating a weaker interaction between task and congruency for the touchscreen response interface than the keyboard response interface.

*Null effect testing.* We also ran a Bayesian model to examine potential null effects. As also observed in the hierarchical logistic regression model, results suggested that the 95% CI for the interaction between task and response interface, congruency, and response interface was outside of zero, indicating that the interaction between task and congruency was less pronounced for the touchscreen version than the keyboard version (see Table 4). However, the main effect of the response interface and all other interactions between the other variables and the response interface indicated that the 95% CI was around zero, favoring a null effect (see Table 4).

### Discussion

The aim of Study 2 was to examine whether we can observe canonical behavioral effects in the Stroop paradigm in touchscreen versus keyboard experiments. The results from both experiment versions were mostly consistent. Both versions revealed a significant congruency effect in RT, and the response interface interactions were not significant, indicating that the touchscreen did not qualitatively change the effects typically found in keyboard versions of the Stroop task. However, we did not find a main effect for the task factor in the touchscreen version while observing a significant effect of the task factor in the keyboard version. Also, we did not find a significant task effect for error rates in both versions. We found a significant influence of the interface on effects typically found in the Stroop task. While the touchscreen version did not show a significant task congruency interaction, the keyboard version did. However, we believe that these inconsistent results are not the result of differences in the interface but rather external factors, like sample size. There were other effects traditionally associated with the Stroop task that were missing in the keyboard version as well, like a task and sequential congruent effect in error rates, also in the keyboard version of the experiment. This might indicate that the low sample size might have led to an underpowered study. Taking together the more conclusive RT results and the error rate results, the findings suggest that the type of response interface did not fundamentally change the effects typically associated with the Stroop task.

## General discussion

This article introduced an open-source touchscreen extension for the jsPsych software for online experiments (de Leeuw, 2015; de Leeuw et al., 2023), which allows researchers to easily build online experiments for touchscreen devices. We tested this touchscreen extension across two experimental paradigms – a perceptual decision-making task based on the random-dot kinematogram and the Stroop task. To evaluate this plugin, we compared the results of these touchscreen versions with results obtained from compatible keyboard versions of the experiments. Results of these experiments suggest that data obtained via touchscreen devices recover key behavioral effects across paradigms and are similar to the effects observed with keyboard devices. Together, results from both studies provide evidence that the developed touchscreen extension can be implemented in online experiments to measure psychometric measures and interference effects.

The use of touchscreen devices for data collection offers a great advantage, as experiments can be conducted almost anywhere and at any time (e.g., Passell et al. (2021)). This makes it easier to collect data for more intricate experimental designs, such as multi-session studies (e.g., Snijder et al. (2023); Singh et al. (2023)), longitudinal studies, or studies that require participants to do the experiment in more realistic settings (e.g., Zech et al. (2022); Singh et al. (2023)).^4^

Our results contribute to a growing body of research comparing data collected via touchscreens with data collected via keyboards in several respects. First, Bignardi et al. (2021) collected data from children (age range: 7-9) on several cognitive tasks with touchscreens, but not on the two tasks tested in this study: the random-dot kinematogram task and the Stroop task. In contrast to Bignardi et al. (2021) who did not compare their touchscreen results with data collected via keyboards, other studies investigated differences between data collected with touchscreens compared to keyboards and reported generally slower response times for data collected with touchscreens (Pronk et al., 2023; Passell et al., 2021). Our results comport with these findings, showing that participants responded slower on the touchscreen versions compared to the desktop versions (see the discussion on limitations below). In another study, Lacroix et al. (2021) reported similar results on visuospatial abilities when comparing data collected via touchscreens with data collected with traditional paper-pencil methods and observed strong correlations between the touchscreen data and the paper-pencil data. Furthermore, Pronk et al. (2023) examined whether the test re-test reliability of interference effects observed from the flanker task differs between touchscreen and keyboard devices collected through a crowd-sourced research platform. They observed no differences in test-retest reliability between device measures. Our findings build on these results by providing further evidence on the construct validity of results when data is collected in the web via touchscreen and keyboard devices. We found similar behavioral effects for the two perceptual decision making. In the first experimental study, we observed that touchscreen and keyboard versions of the same experiment yield similar psychometric curves for reaction times and error rates in a perceptual decision-making task. We also observed similar behavioral metrics between the touchscreen and keyboard versions in our second study of the Stroop task. In particular, we observed that the behavioral effects commonly observed in the Stroop paradigm, such as the congruency effect, the Stroop effect, and the sequential congruency effect, were similar between both response platforms.

The development of this touchscreen extension expands the possibilities of collecting data via online experiments using jsPsych software. This involves the implementation of arbitrary response buttons in terms of graphics and colors. While this has not been the focus of this study, the design of the random-dot kinematogram experiment hints at these possibilities by displaying an arrow on the touchscreen button that indicates the associated response. The plugin would enable new experiments that cannot be implemented in keyboard experiments, such as manipulating the spatial overlap between stimuli and response buttons.

A limitation of the touchscreen extension may be generally slower responses that possibly result from the nature of the input device and their operating systems. Comporting with our findings, previous studies reported slower response times for data collected via smartphones, in particular Android devices, compared to keyboard devices (Passell et al., 2021). There is, of course, variability across devices. Nicosia et al. (2023) analyzed the timing accuracy of 26 different smartphones and observed considerable variability in general response times between smartphone devices and their operating systems (ranging between 35 to 140ms). Despite the consistent finding that response times are generally slower on smartphone touchscreens compared to standard keyboards, it remains to be determined whether the extension code itself contributes significantly to the response latency. To yield more comparable results with traditional input devices, researchers may want to consider to restrict their data collection to touchscreen devices with low response latencies and/or match device types across groups. Another factor that may have caused slower response times in our study are different browsers from which the data was collected (Pronk et al., 2020). In addition, devices vary in screen size-a factor that has been shown to impact overall response times (Passell et al., 2021). In an exploratory analysis, we tested whether our anticipated effects were further modulated by screen size but found no evidence for such a modulatory effect. However, this may be due to the relatively small sample sizes used in our study which were estimated to detect the canonical behavioral effects of perceptual decision-making tasks. Nevertheless, future research collecting data through crowdsourcing research platforms are well advised to control for device type, operating system, browser, and screen size.

Despite not controlling for device type, operating system, browser, and screen size, we observed similar canonical behavioral effects for both perceptual decision making tasks across touchscreen and keyboard devices. While the primary purpose of our study was to introduce the touchscreen extension and the evaluation of this extension was only of secondary nature, an interesting future research question may be to systematically test whether different sources known to affect general response times (i.e., device type, operating system, browsers, and screen size) also affect psychometric measures and interference effects.

In sum, this touchscreen extension expands the possibilities for conducting online research in the behavioral sciences via the jsPsych software. We hope that this extension will enable new forms of data collection and that it will aid in overcoming practical obstacles associated with data collection from traditional personal computers.

## Supplementary Information

Below is the link to the electronic supplementary material.

Supplementary file 1 (docx 419 KB)

## Data Availability

The data and all data analysis scripts are available on https://osf.io/v8n3x/?view_only=68ed5f56aed44167b850bfad2159abfd.

## Footnotes

1 The brightness sensors quantified stimulus onset, whereas the solenoid documented the touchscreen response of the participant.

2 Note that the touchscreen extension requires the jsPsych 7 JavaScript library (https://www.jspsych.org/).

3 Note that effect sizes are typically simulated for hierarchical regression models if prior data exists.

4 We acknowledge that online experiments on mobile phones and tablets could also be conducted without the touchscreen extension by responding to stimuli via a keyboard that pops up on the touchscreen device. However, such a keyboard on the touchscreen would have the disadvantage of using up relatively large proportions of screen space. In addition, the smaller precision obtained from virtual keys on a keyboard (compared to virtual buttons) may lead to a larger rate of erroneous or slow responses and, consequently, to a more unsatisfying user experience.

## Code Availability

The touchscreen plugin is fully available, documented, and free to use on https://github.com/jspsych/jspsych-contrib/tree/main/packages/extension-touchscreen-buttons.
