# Supplementary material for: A jsPsych touchscreen extension for behavioral research on touch-enabled interfaces
Source: Behav Res Methods. 2024 Jul 12;56(7):7814–30. doi: 10.3758/s13428-024-02454-9 (PMC11549123; doi:10.3758/s13428-024-02454-9)
Supplement: Supplementary file 1 — (docx 419 KB) [file 13428_2024_2454_MOESM1_ESM.docx]

**Supplementary Material**


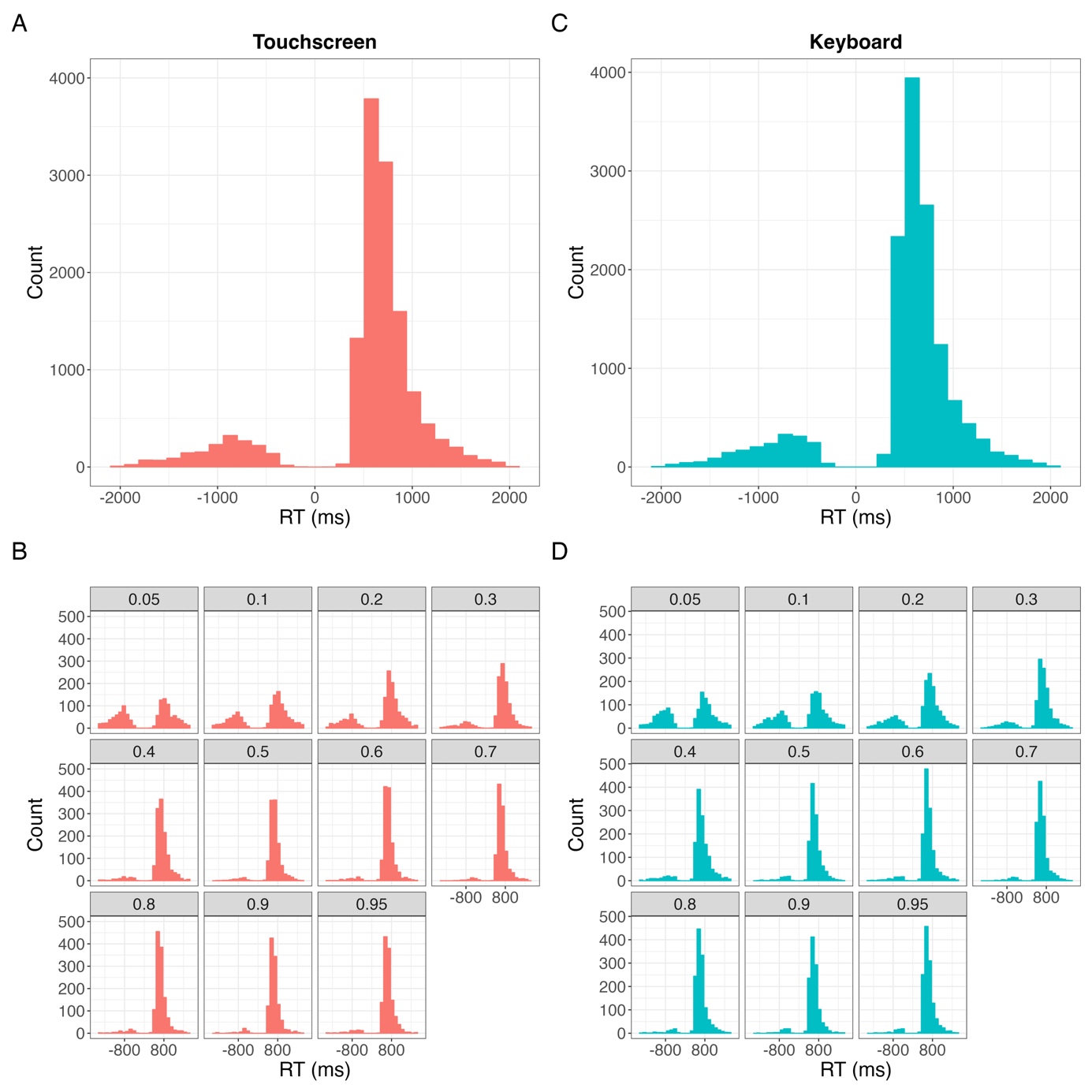


**S1 RT Histogramm for the touchscreen (left panel) and keyboard (right panel) experiment version.** Upper row: Distribution of all incorrect (negative) and correct (positive) RTs for the touchscreen (A) and keyboard (B) experiment version. Bottom row: RT distributions for each coherence level.

**Study 1**

**Response Interface and Screen Size Effects**

We briefly report the main effects in the text below and additionally list all results in Table S2.

***Reaction Times***

Results of the hierarchical linear regression model indicated a significant main effect for the coherence term (*b* = -0.24; *t* = -2.21; *p* = .03). The main effect for the response interface term was not significant (*b* = -0.02; *t* = -0.11; *p* = .911). The main effect for the screen size term was not significant (*b* < -0.01; *t* = -0.71; *p* = .482). None of the possible interactions between these three terms were significant.

***Accuracy***

Results of the hierarchical linear regression model indicated a significant main effect for the coherence term (*b* = 5.05; *z* = 3.72; *p* < .001). The main effect for the response interface term was not significant (*b* = 0.43; *z* = 1.14; *p* = .253). The main effect for the screen size term was not significant (*b* < 0.01; *z* = 1.83; *p* = .067). None of the possible interactions between these three terms were significant.

**Table S2**

| **Random-Dot Kinematogram Task** | | | | | | | | |
| --- | --- | --- | --- | --- | --- | --- | --- | --- |
|  | **log(RT)** | | | | **Accuracy** | | | |
| *Coeffcient* | *b* | *SE* | *t* | *p* | *b* | *SE* | *z* | *p* |
| (Intercept) | 6.87 | 0.15 | 46.34 | **<0.001** | -0.26 | 0.33 | -0.78 | 0.434 |
| Coherence | -0.24 | 0.11 | -2.21 | **0.027** | 5.06 | 1.36 | 3.72 | **<0.001** |
| Response Interface | -0.02 | 0.17 | -0.11 | 0.911 | 0.43 | 0.38 | 1.14 | 0.253 |
| Screen Size | -0.00 | 0.00 | -0.71 | 0.480 | 0.00 | 0.00 | 1.83 | 0.067 |
| Coherence:Response Interface | -0.01 | 0.13 | -0.10 | 0.921 | -0.49 | 1.56 | -0.31 | 0.754 |
| Coherence:Screen Size | -0.00 | 0.00 | -1.15 | 0.251 | -0.00 | 0.00 | -0.43 | 0.665 |
| Response Interface:Screen Size | -0.00 | 0.00 | -0.10 | 0.924 | -0.00 | 0.00 | -1.40 | 0.161 |
| Coherence:Response Interface:Screen Size | 0.00 | 0.00 | 0.67 | 0.500 | 0.00 | 0.00 | 0.24 | 0.807 |
|  |  |  |  |  |  |  |  |  |

**
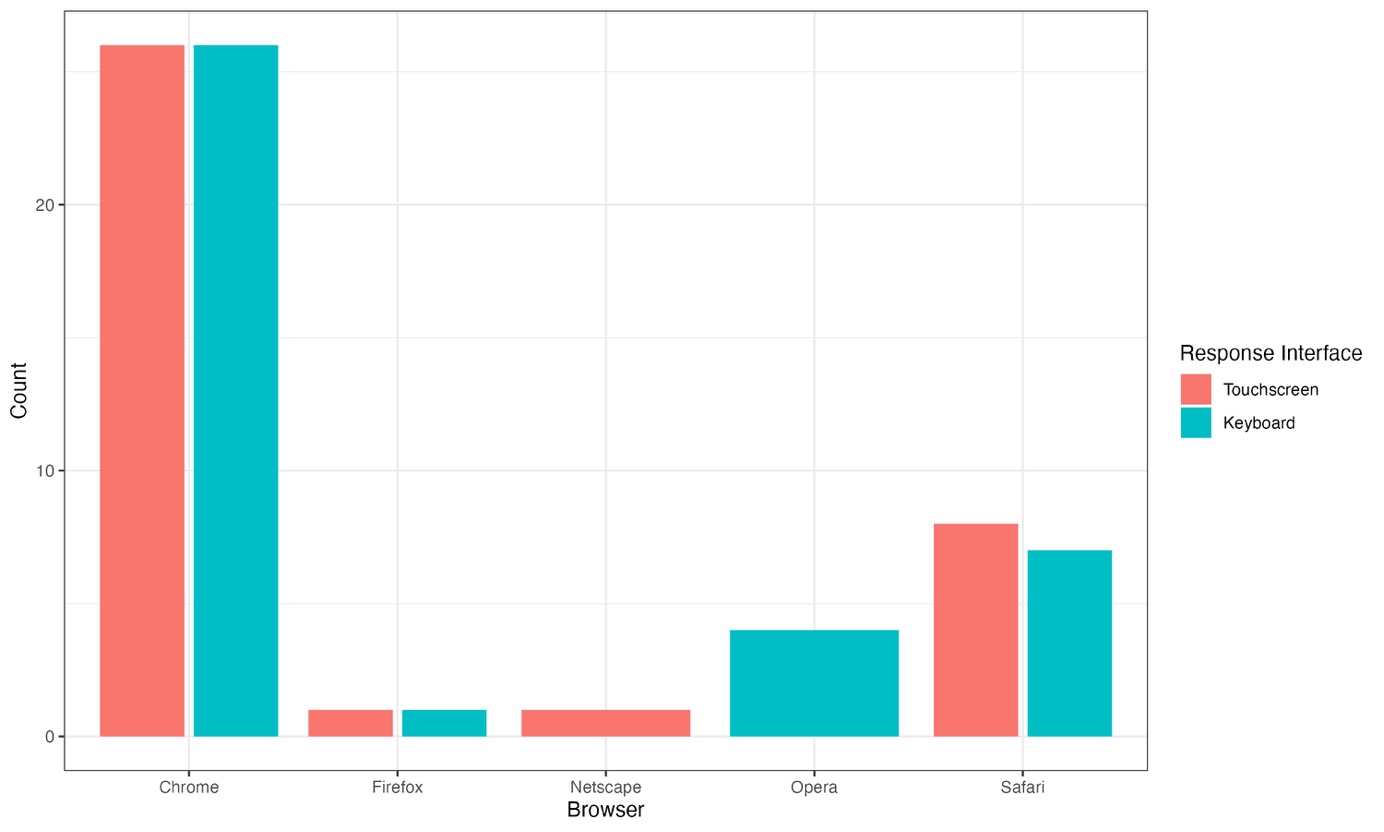
**

**S4 Histogram of browsers participants used for Experiment 1 for the touchscreen version and keyboard version.**
